# UAV propeller fault diagnosis using deep learning of non-traditional χ^2^-selected Taguchi method-tested Lempel–Ziv complexity and Teager–Kaiser energy features

**DOI:** 10.1038/s41598-024-69462-9

**Published:** 2024-08-10

**Authors:** Luttfi A. Al-Haddad, Wojciech Giernacki, Ali Basem, Zeashan Hameed Khan, Alaa Abdulhady Jaber, Sinan A. Al-Haddad

**Affiliations:** 1 Training and Workshops Center, University of Technology- Iraq, Baghdad, Iraq; 2 Faculty of Automatic Control, Robotics and Electrical Engineering, Institute of Robotics and Machine Intelligence, Poznan University of Technology, Poznań, Poland; 3 Air Conditioning Engineering Department, Faculty of Engineering, Warith Al-Anbiyaa University, Karbala, Iraq; 4 Interdisciplinary Research Center for Intelligent Manufacturing & Robotics (IRC-IMR), King Fahd University of Petroleum & Minerals (KFUPM), 31261 Dhahran, Saudi Arabia; 5 Mechanical Engineering Department, University of Technology- Iraq, Baghdad, Iraq; 6 Civil Engineering Department, University of Technology- Iraq, Baghdad, Iraq

**Keywords:** UAV, Fault diagnosis, Artificial intelligence, Permutation entropy, Lempel–Ziv complexity, Teager–Kaiser energy operator, Aerospace engineering, Electrical and electronic engineering

## Abstract

Fault detection and isolation in unmanned aerial vehicle (UAV) propellers are critical for operational safety and efficiency. Most existing fault diagnosis techniques rely basically on traditional statistical-based methods that necessitate better approaches. This study explores the application of untraditional feature extraction methodologies, namely Permutation Entropy (PE), Lempel–Ziv Complexity (LZC), and Teager–Kaiser Energy Operator (TKEO), on the PADRE dataset, which encapsulates various rotor fault configurations. The extracted features were subjected to a Chi-Square (χ^2^) feature selection process to identify the most significant features for input into a Deep Neural Network. The Taguchi method was utilized to test the performance of the recorded features, correspondingly. Performance metrics, including Accuracy, F1-Score, Precision, and Recall, were employed to evaluate the model’s effectiveness before and after the feature selection. The achieved accuracy has increased by 0.9% when compared with results utilizing traditional statistical methods. Comparative analysis with prior research reveals that the proposed untraditional features surpass traditional methods in diagnosing UAV propeller faults. It resulted in improved performance metrics with Accuracy, F1-Score, Precision, and Recall reaching 99.6%, 99.5%, 99.5%, and 99.5%, respectively. The results suggest promising directions for future research in UAV maintenance and safety protocols.

## Introduction

### Scope and overview

Unmanned Aerial Vehicles (UAVs), so-called and mostly referred to as drones, have evolved significantly over the past decade^1–3^. UAVs transformed from niche military assets to ubiquitous tools in civilian sectors^4,5^. Initially deployed for aerial reconnaissance and remote sensing, UAVs now play pivotal roles in diverse applications ranging from disaster response and environmental monitoring to urban planning and delivery services^6^. The advancement in UAV technologies has been bolstered by innovations in communication systems, sensor design, and computational capabilities, enabling more complex and varied operations^7^.

The broadening scope of UAV applications has underscored the need for robust, efficient, and fail-safe operational technologies, especially in critical components such as propellers^8,9^. Propellers, essential for the maneuverability and stability of UAVs, are prone to faults that can significantly impair functionality and safety^10–13^. Traditional fault diagnosis methods, typically reliant on straightforward statistical tools, often fall short in addressing the complex nature of UAV operational environments and the subtle manifestations of such faults^11,14^.

This paper introduces an innovative approach to UAV propeller fault diagnosis by leveraging non-traditional features—specifically Permutation Entropy (PE)^15^, Lempel-Ziv Complexity (LZC)^16^, and Teager–Kaiser Energy Operators (TKEO)^17^—integrated with a Deep Learning framework^18^. Additionally, the paper also presents the significance of these features when tested by the Taguchi methodology. This method capitalizes on the sophisticated capabilities of these features to extract meaningful patterns from the noise-dominated data typical of UAV operations. The utilization of the PADRE—Propeller Anomaly Data REpository enhances the reliability of the fault detection process as it offers a rich dataset that encapsulates various UAV rotor fault configurations^19–21^.

### State-of-the-art

The field of UAV fault diagnosis has witnessed considerable advancements through various methodologies as summarized and presented in Table 1. These studies predominantly focus on feature extraction and classification techniques as they employ methods ranging from statistical analysis to advanced machine learning algorithms. Notably, recent works have leveraged acoustic and vibration data and embedded sensors to enhance fault detection capabilities. However, while these approaches offer valuable insights, they typically do not explore the integration of multiple, diverse non-traditional features in conjunction with deep learning techniques. This gap underscores the novelty of the current research, which utilizes a combination of complex untraditional features. These methodologies are synthesized in a deep learning framework to advance the precision and reliability of UAV propeller fault diagnosis which sets the stage for the identification of research gaps and novelties next.

**Table 1:** Commonly employed methodologies for UAVs fault detection and diagnosis.

References	Methodology	Remarks
^22^	Statistical feature extraction in UAV motors	Focuses on sound-based methods, lacks integration with deep learning
^23^	SVM classification with embedded feature extraction	Limited to specific motor faults, does not utilize complex feature sets
^24^	Convolutional neural networks with transfer learning for audio-based diagnosis	Employs CNNs but does not combine with other sophisticated methods
^25^	Compound fault labeling and diagnosis based on flight data and BIT record	Integrates multiple data sources but lacks advanced analytical techniques
^26^	Audio signal analysis for unbalanced blade detection	Utilizes traditional signal processing, limited in scope to specific fault types
^27^	Acoustic inspection system for wind turbines via UAVs	Focuses on structural health, not directly applicable to UAV internal faults
^28^	Frequency domain analysis for wind turbine inspection	Specific to wind turbines, does not translate directly to UAVs
^29^	Data-driven diagnosis under multiple operation conditions	Emphasizes data-driven methods without integrating novel feature extraction
^30^	Robust adaptive sliding mode for fault-tolerant control	Focuses on control solutions rather than diagnostic capabilities
^31^	Sensor fault diagnosis with Auto Sequential Random Forest	Utilizes advanced forestry methods but does not integrate diverse data features

Following the summary presented earlier, it’s evident that a variety of methodologies have been employed in the field of UAV fault diagnosis. Altinors, Yol, and Yaman utilized statistical feature extraction to detect UAV motor faults using acoustic signals, demonstrating initial steps toward sound-based diagnostic methods^22^. Yaman, Yol, and Altinors further explored this avenue, integrating embedded feature extraction with SVM classification specifically tailored for UAV motors^23^. Liu, Chen, and Zheng applied convolutional neural networks of transfer learning to harness audio data to diagnose quadrotor faults, yet their application remained singularly focused on one type of data^24^. Zheng et al.^25^ introduced a compound fault labeling and diagnosis method that relies on an integration of flight data and built-in test records with limited analytical depth. Moreover, Rangel-Magdaleno et al.^26^ detected unbalanced UAV blades using audio signals, focusing on a specific fault type which restricts broader application possibilities. García Márquez, Bernalte Sánchez, and Segovia Ramírez developed an acoustic inspection system tailored for wind turbines, indicating the versatility of UAV applications but not addressing UAV internal diagnostics^27^. Similarly, Sánchez, Ramirez, and Márquez employed sound frequency domain analysis in their inspections of wind turbines^28^. Adding up, Liang and colleagues advanced a data-driven approach that contemplates multiple operational conditions of fixed-wing UAVs, yet they did not integrate novel sensor technologies or complex feature analyses^29^. Nguyen and Hong focused on fault-tolerant control strategies using a robust adaptive sliding mode, shifting the emphasis from diagnostic to control methodologies^30^. Lastly, Ai, Song, Cai, and Zhao applied an Auto Sequential Random Forest for diagnosing sensor faults in quadrotors, innovating with machine learning techniques that enhance fault detection accuracy, though without exploring a fusion of different data types^31^.

### Research gap and novelties

Despite the advancements in prior studies, a research gap exists in the integrated use of complex, untraditional features for UAV fault diagnosis. Most existing methodologies focus on conventional statistical analyses or basic machine learning techniques that may not fully capture the nuanced dynamics of UAV propeller systems. Additionally, the potential of combining various non-traditional features using advanced computational models has not been thoroughly explored. This gap indicates a need for innovative approaches that leverage the complexity of new feature types, specifically within the context of vibration analysis, to enhance diagnostic accuracy and reliability. The current study addresses these challenges by introducing a novel methodology that not only enriches the analytical landscape but also enhances the practical capabilities of UAV fault diagnostics, thereby setting a new benchmark in the field. The contributions of the current research can be minimized in three basic points as follows:

- Introduction of complex, untraditional vibration analysis techniques: This research utilizes new features such as PE, LZC, and TKEO to provide a more detailed and accurate fault analysis of UAV propellers.
- Application of deep learning to modern feature sets: By integrating these features into a Deep Neural Network (DNN) framework, the study exploits the power of modern artificial intelligence to significantly improve the effectiveness of fault detection in UAVs.
- Employment of Chi-Square (χ^2^) for optimized feature selection: The study employs the Chi-Square statistical test to meticulously select the most impactful features to ensure that the DNN is trained on the most relevant and significant data.
- The application of a testing approach that depends on the Taguchi method, to collectively demonstrate the dependability of each of the selected feature for such diagnosis scheme.

The remainder of this paper is structured to systematically unfold the research methodology and findings as follows: Section “Experimental work” describes the experimental work using the PADRE dataset with its procedures. Section “Advanced state-of-the-art selected-tested features” introduces the proposed pattern triggering attributes and discusses the process of important feature selection. Section “Deep learning” details the deep learning model’s architecture and its integration with the selected features. Section “Results and discussion” presents a comprehensive analysis of the experimental results while comparing them with existing methods. Finally, Section “Conclusions and future directions” summarizes the contributions and explores potential avenues for further research.

## Experimental work

### Methodology

**Figure 1:**
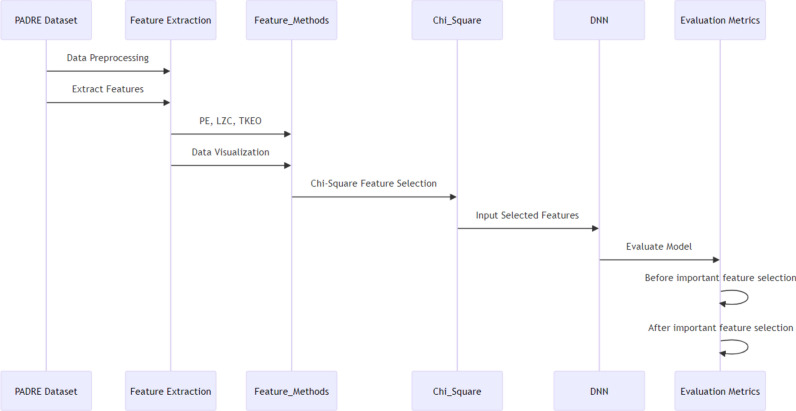
Sequence diagram for the proposed methodology.

The methodology employed in this study is systematically illustrated in Figure 1. Initially, the PADRE Dataset is utilized for both data preprocessing and feature extraction, where essential features are derived. The process is to be explained in the upcoming subsection. The feature extraction stage applies advanced methods including PE, LZC, and TKEO, supplemented by data visualization to aid in the interpretability of these features. Following this, the Chi-Square test is employed to select the most significant features, which are then inputted into a DNN. The performance of the DNN is evaluated using a set of metrics, both before and after the application of important feature selection. This sequence ensures a rigorous approach to UAV propeller fault diagnosis by integrating advanced feature extraction methods with sophisticated DNN to enhance diagnostic accuracy.

### PADRE and data acquisition procedures

The PADRE dataset was engineered to facilitate comprehensive UAV fault detection research as it provides a structured compilation of sensor data from UAVs under various fault conditions^21^. This dataset is crucial for testing and developing advanced fault detection methodologies due to its rich, detailed, and versatile nature.

**Figure 2:**
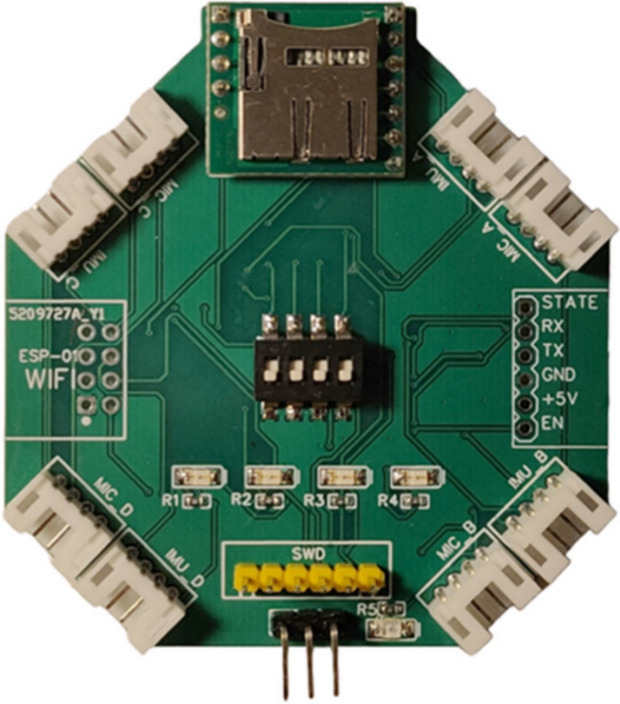
Top layer of the custom data acquisition PCB^21^.

A custom-designed data acquisition system was developed to capture faults in UAV drive units. Recognizing the limitations of standard UAV sensors, which often feature low sampling rates and restricted access, a high-performance system was crafted to ensure precise fault localization and classification. This system is based upon the STM32H743IIT6 microcontroller, equipped with an array of passive components for robust performance in real-time data processing and fault detection^21^. The system’s design ensures it is lightweight and compact to make sure that the process minimizes any impact on UAV dynamics. It features a 4-position DIP switch that aids in quick data classification and simplifies the post-processing stages, critical for real-time fault analysis. The system’s layout and major components are illustrated in Figure 2.

**Figure 3:**
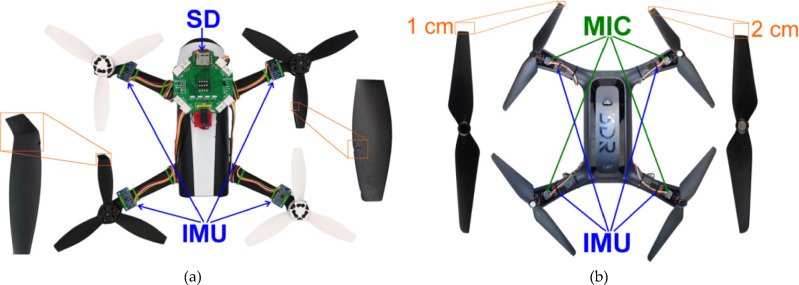
Sensor configurations and fault indications on UAVs^21^: (**a**) Parrot Bebop 2; (**b**) 3DR Solo.

The system was tested on two distinct UAV models: the Parrot Bebop 2 and the 3DR Solo, both chosen for their representative design and common usage in UAV research, as stated in the previously published experimental work^21^. The Bebop 2 and Solo differ significantly in terms of physical dimensions, operational capabilities, and sensor configurations, making them ideal for demonstrating the versatility of the data acquisition system. The key specifications of these UAVs are outlined in Table 2, for general information. For the Parrot Bebop 2, data were collected over 20 flights, each precisely timed to gather comprehensive sensory feedback across various fault conditions, including chipped edges and bent propeller tips. For the 3DR Solo, nine flights were conducted to simulate different degrees of propeller tip losses. These experiments were meticulously recorded, producing a vast array of data points per flight, which were then stored and categorized for efficient retrieval and analysis. A sophisticated arrangement of accelerometers, gyroscopes, barometers, and microphones was employed to capture a broad spectrum of fault-related data. This setup allowed for the detailed monitoring of each drone’s operational status during flight, ensuring that any deviations from normal operation were accurately logged. The configuration of sensor sets and the type of data collected during the experiments with both UAV models are depicted in Figure 3. Moreover, Table 3 details the technical parameters and properties of the inertial sensors used, which includes their sensitivity and data output rates to provide a much more deeper understanding of the data’s granularity and the system’s capacity to detect subtle variations indicative of faults.

**Table 2:** IMU modules parameters^21^.

UAV Model	Dimensions (mm)	Weight (g)	Propeller size (cm)	Battery capacity (mAh)	Flight time (minutes)	Maximum speed (m/s)	WiFi range (m)
Parrot Bebop 2	328 × 328 × 89	500	15.2	2700	Up to 25	16	300
3DR Solo	250 × 250 × 260	1500	25	5200	Up to 25	24.5	800

**Table 3:** Specifications of IMU modules used in experiments^21^.

Parameters	Accelerometers	Gyroscope
ADC word length	16 bit	16 bit
Number of axis	3	3
Range	$\pm 16$ g	$\pm 2000$ dps
Sensitivity	2048–16384 LSB/g	16.4–131 LSB/dps
Output data rate	4000 Hz	8000 Hz

**Figure 4:**
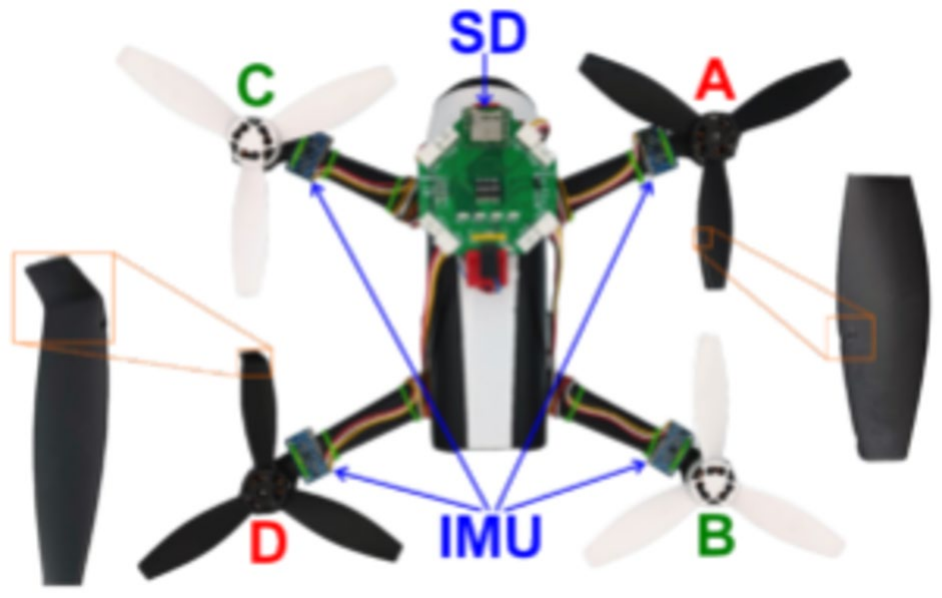
Propeller labelling for fault classification^21^.

**Figure 5:**
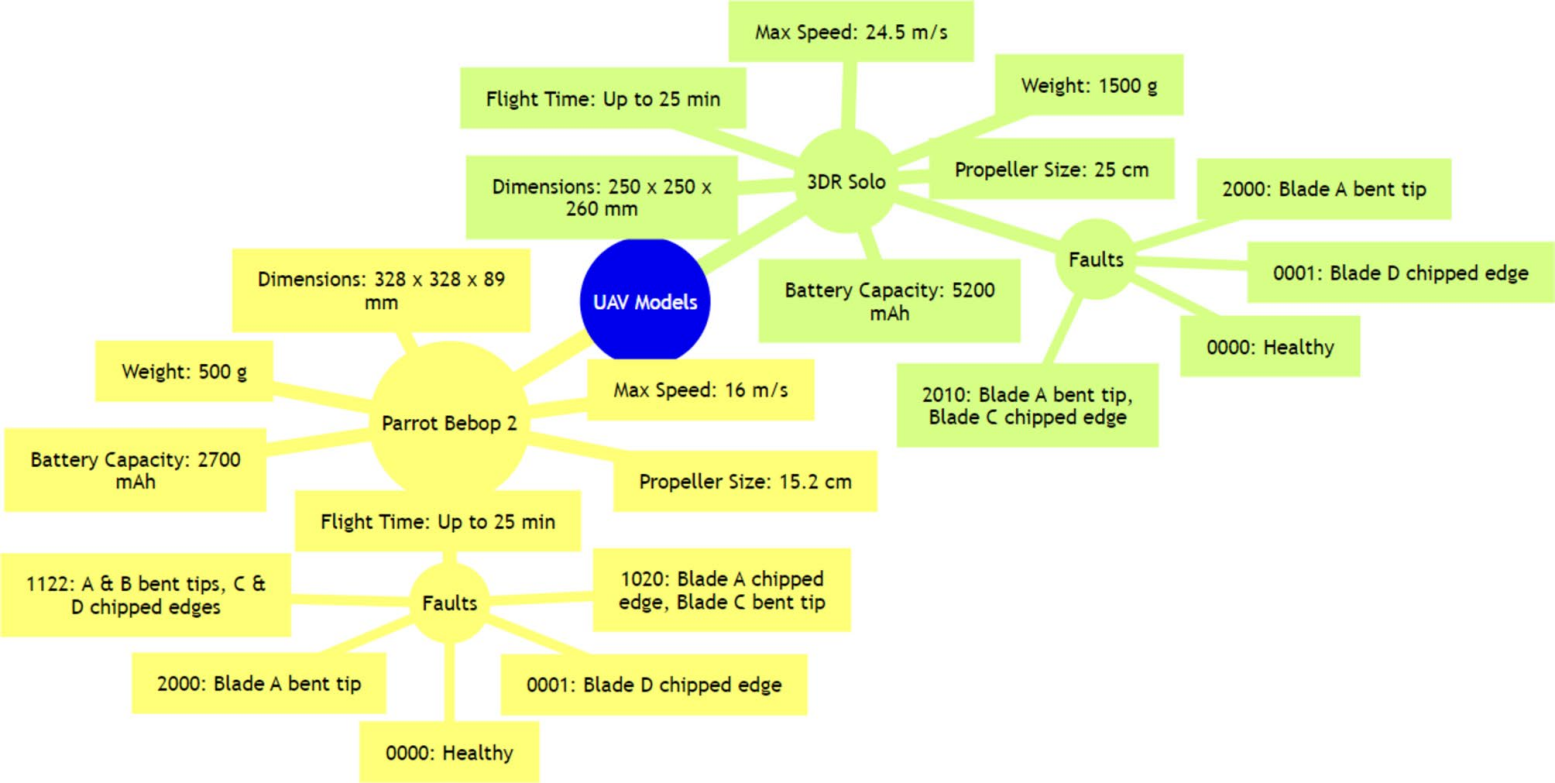
Mind-map diagram for the selected drones.

The integration of this data acquisition system with real-time processing and AI-based fault classification showcases the potential for significant advancements in UAV fault diagnostical schemes. The collected data underpin the development of sophisticated fault detection algorithms that leverage the detailed inputs from the diverse sensor array. This approach enhances fault classification and detection accuracy in addition to the fact that it contributes to the broader application of UAVs in complex operational environments. The custom-designed data acquisition system and its detailed components are visually outlined in Figure 4, which provides an illustrative overview of the sensor placement and PCB configuration used in the study. This visualization aids in understanding the technical setup that facilitated the high-fidelity data collection critical for fault analysis. The specific fault scenarios and the corresponding data files used for the analysis are systematically cataloged in Table 4, where this table enumerates the various fault conditions simulated in the experiments with the Parrot Bebop 2 and 3DR Solo drones by detailing the precise fault type and affected propellers. Figure 5 depicts the overall mind-map diagram for the selected two operational drones of the study.

**Table 4:** Data files and corresponding fault scenarios with specific fault types^21^.

UAV model	Identifier	Fault description
Parrot Bebop 2	0000	All propellers functional
	0001	Single propeller fault: Propeller D with a chipped edge (type 1)
	1020	Two propeller faults: Propeller A with a chipped edge, Propeller C with a bent propeller tip (types 1 and 2)
	1122	All propellers with faults: Propellers A and B with bent propeller tips, Propellers C and D with chipped edges (types 2 and 1)
	2000	Single propeller fault: Propeller A with a bent propeller tip (type 2)
3DR Solo	0000	All propellers functional
	0001	Single propeller fault: Propeller D with a chipped edge (type 1)
	2000	Single propeller fault: Propeller A with a bent propeller tip (type 2)
	2010	Two propeller faults: Propeller A with a bent propeller tip and Propeller C with a chipped edge (types 2 and 1)

## Advanced state-of-the-art selected-tested features

### Proposed pattern triggering attributes

The use of advanced feature analysis sets new foundations for the enhancement of AI-related methodologies in variant applications^32^. Permutation Entropy (PE) is a measure of complexity that evaluates the order relations between values in a time series. It provides insights into the underlying dynamics of the data by analyzing the frequency of different ordinal patterns. This formula can be visually expressed as Eq. (1) states:

1 $$PE= -{\sum }_{\pi }p\left(\pi \right)\times \text{log}(p\left(\pi \right)),$$

where $\pi$ is the permutation pattern of length *m* in a signal and $p\left(\pi \right)$ is the probability of the permutation pattern, which was stated earlier. Herewith, PE is used on acceleration time-domain signals from accelerometers and degree recordings from gyroscopes to measure the complexity and regularity of the data. By evaluating the order relations between consecutive values, PE helps in identifying changes in the dynamics of the UAV, such as transitions between different flight modes or the onset of faults. High permutation entropy indicates more randomness and potential anomalies, while lower values suggest more predictable patterns, which can be crucial for monitoring the UAV’s health and detecting faults early.

Lempel-Ziv Complexity (LZC) quantifies the complexity of a binary sequence derived from a time series. It is based on the number of distinct substrings and their frequency of occurrence. This measure captures the randomness and structure within the data and can be expressed by Eq. (2):

2 $$LZC= \frac{C(n)}{n/\text{log}(n)},$$

where $C(n)$ is the number of distinct substrings (or complexity count) encountered in the binary sequence, $n$ is the length of the binary sequence, $\text{log}(n)$ is the natural logarithm of the length of the sequence. In this paper, LZC is applied to the binary sequences derived from the acceleration signals of accelerometers and angular velocity data of gyroscopes to quantify the complexity and structure of these signals. By converting the time-series data into binary sequences and measuring the number of distinct substrings, LZC captures the inherent randomness and patterns in the UAV’s motion. Higher complexity can indicate irregular movements or faults, whereas lower complexity suggests more stable and predictable flight. This measure helps in identifying abnormal behavior in the UAV, which is essential for timely maintenance and fault diagnosis.

Teager–Kaiser Energy Operator (TKEO) measures the instantaneous energy of a time series. It is described in Eq. (3) below and it is particularly useful for detecting and characterizing transient events within the signal.

3 $$\Psi \left[x\left(t\right)\right]= {x(t)}^{2}-x\left(t-1\right)\times x\left(t+1\right),$$

where $x(t)$ is the value of the time series at time $t$, $x(t-1)$ is the value of the time series at time $t-1$, $x(t+1)$ is the value of the time series at time $t+1$. TKEO is used on the time-domain signals of accelerometers and gyroscopes of the current work to measure the instantaneous energy of the signals. TKEO is particularly effective in detecting transient events, such as sudden changes in acceleration or angular velocity, which may indicate faults or impacts. By calculating the energy at each point in the time series, TKEO provides a detailed view of the signal’s dynamics, helping in the identification of abrupt changes or anomalies that could compromise the UAV’s performance. This real-time energy analysis is crucial for monitoring the UAV’s condition and ensuring safe operation.

### Important feature selection

Chi-square (χ^2^) feature selection is a statistical method used to determine the significance of individual features in relation to a target variable, typically in classification tasks. It evaluates whether the observed frequency distribution of a feature differs significantly from the expected distribution. This method helps in identifying the most relevant features for model training, enhancing the performance of the classifier by reducing dimensionality and improving generalization. Equation (4) elaborates on the mathematical formula for χ^2^, as follows:

4 $${\upchi }^{2 }=\sum_{j=1}^{N}\frac{{\left({Y}_{j}-{u}_{j}\right)}^{2}}{{u}_{j}}$$

where $N$ is the number of observed events or categories, ${Y}_{j}$ represents the observed frequency for the jth category or event, and ${u}_{j}$ is the expected frequency for the jth category or event, under the null hypothesis that there is no association between the feature and the target variable. In the present work, applying chi-square feature selection helps in identifying the most informative features from the PE, LZC, and TKEO calculations. By selecting features that have the highest chi-square scores, the method ensures that the selected features are statistically significant in distinguishing between normal and faulty conditions, thereby it improves the efficiency and accuracy of the fault detection model.

### Taguchi method

In order to further test the dependability of each of the three features, the study leverages the Taguchi method, which is an arising state-of-the-art approach that was adopted recently^33,34^. The approach employs a structured series of experiments using an orthogonal array, which methodically explores different combinations of variables at specified levels. The effectiveness of each experimental configuration is measured by the Signal-to-Noise (S/N) ratio. This particular study uses the “larger is better” variant of the S/N ratio, as detailed in Eq. (5) found below^35^:

5 $$\text{SN}=-10\text{log}\frac{1}{\text{n}}\sum_{i=1}^{N} \frac{1}{{y}^{2}}$$

where *y* represents the performance metric (e.g., accuracy) and *n* denotes the number of trials.

## Deep learning

Deep learning is a subset of machine learning that utilizes neural networks with many layers to model complex patterns in data. It has revolutionized numerous fields by enabling models that automatically learn to make decisions from big data without explicit programming. Its applications span image and speech recognition, autonomous driving, and natural language processing which reflects its versatility and capability to tackle problems that were once considered beyond the reach of automated systems^36–40^. In the domain of UAVs, deep learning has been instrumental in enhancing fault diagnosis systems that allowed for more accurate predictions and robust analysis by processing complex datasets like the PADRE dataset used in UAV propeller fault diagnosis. The neural network employed in this study consists of three hidden layers, utilizing the hyperbolic tangent function *G* as the activation function, the output *y* of the network, and the network weights update using the gradient descent method, collectively defined by the following equations:

6 $$G=\text{tanh}\left(\text{x}\right)= \frac{\text{sinh}\left(\text{x}\right)}{\text{cosh}\left(\text{x}\right)} = \frac{{\text{e}}^{x} - {\text{e}}^{-x}}{{\text{ e}}^{x} + {\text{e}}^{-x}},$$

7 $$y=G\left({\sum }_{i=1}^{n}{w}_{i}{x}_{i}+b\right),$$

8 $$w_{new} = w_{old} - \eta \nabla Q\left( {w_{old} ,x_{i} ,y_{i} } \right),$$

**Figure 6:**
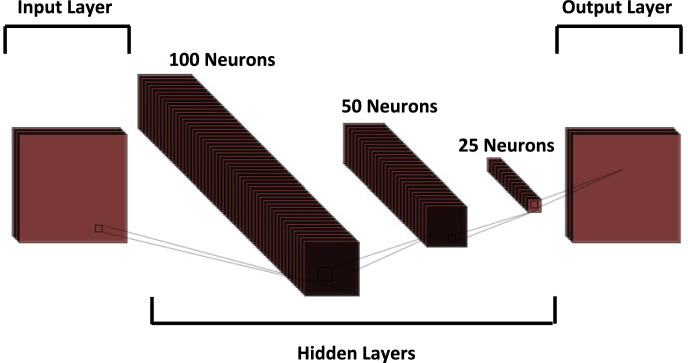
Stacked layers of the DNN.

In the model equations, ${x}_{i}$ represents input features, ${w}_{i}$ are the weights, and *b* is the bias. The parameter η is the learning rate, dictating the step size during the gradient descent optimization. $\nabla Q$ denotes the gradient of the loss function with respect to the weights, guiding how the weights are updated to minimize the loss and improve the model’s prediction accuracy. This framework allows the deep learning model to iteratively learn from the UAV dataset, optimizing performance for fault detection tasks. Figure 6 shows the stacking of the layers utilized in the current DNN and Table 5 summarizes the parameters and their used values for the deep learning model in the current study.

**Table 5:** Utilized DNN parameters.

Parameter	Value
Number of hidden layers	3
Number of neurons in first hidden layer	100
Number of neurons in second hidden layer	50
Number of neurons in third hidden layer	25
Activation function	Hyperbolic Tangent (tanh)
Solver	Adam (adaptive moment estimation)
Number of iterations	1000
Learning rate	0.001
Batch size	32
Loss function	Cross-entropy
Regularization technique	L2 regularization
Initialization method	He initialization

## Results and discussion

### Time-domain and feature-based visualization

**Figure 7:**
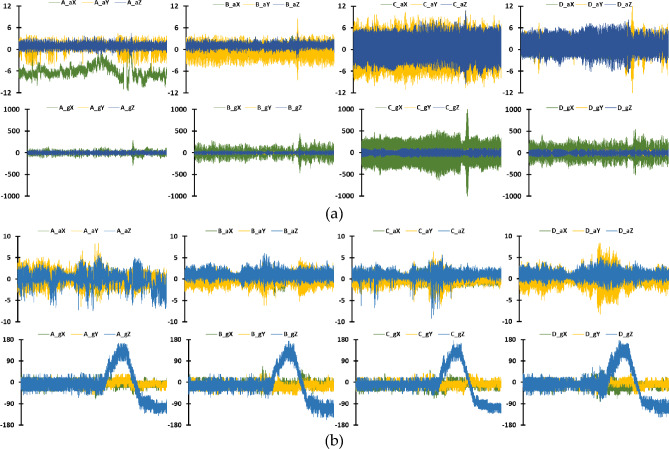
Time-domain recordings of accelerometer and gyroscope data for healthy UAV operation, data were captured over one second, with accelerometer readings in g (ranging from − 12 to 12) and gyroscope readings in dps (ranging from − 1000 to 1000): (**a**) Parrot Bebop 2; (**b**) 3DR Solo.

The UAV is configured with four actuators, labeled as ABCD, each corresponding to a specific propeller. Figure 7 meticulously presents the recordings from accelerometers and gyroscopes associated with these actuators, arranged sequentially from left to right, representing propellers A through D. For the Parrot Bebop 2 UAV, depicted in Figure 7a, the data capture three-dimensional accelerations (x, y, and z axes) over a one-second interval, illustrating the dynamic behavior of each propeller in a healthy state. Similarly, Figure 7b shows the vibrational data for the 3DR Solo UAV, following the same format. Each set of graphs captures the intricate variations and nuances in the data, highlighting slight operational discrepancies even under normal conditions. These differences, while subtle, are critical for demonstrating the rich variability within the PADRE dataset, which challenges and validates the effectiveness of the advanced, non-traditional feature extraction techniques used in this study.

**Figure 8:**
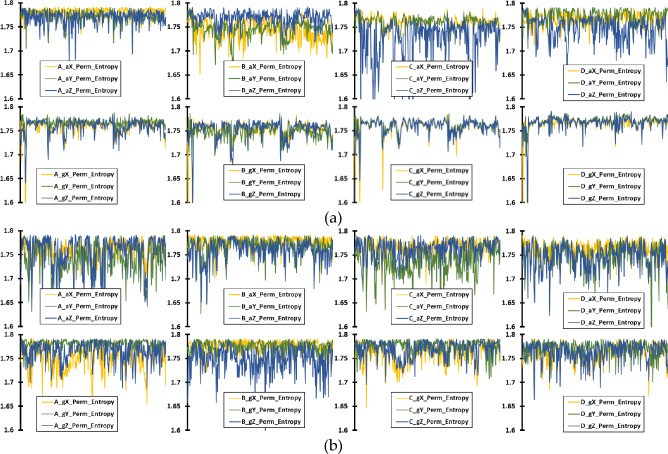
Time-domain PE feature of accelerometer and gyroscope data for healthy UAV operation, with 345 instances in the x axis corresponding for an overall operation of 1 s, for: (**a**) Parrot Bebop 2; (**b**) 3DR Solo.

**Figure 9:**
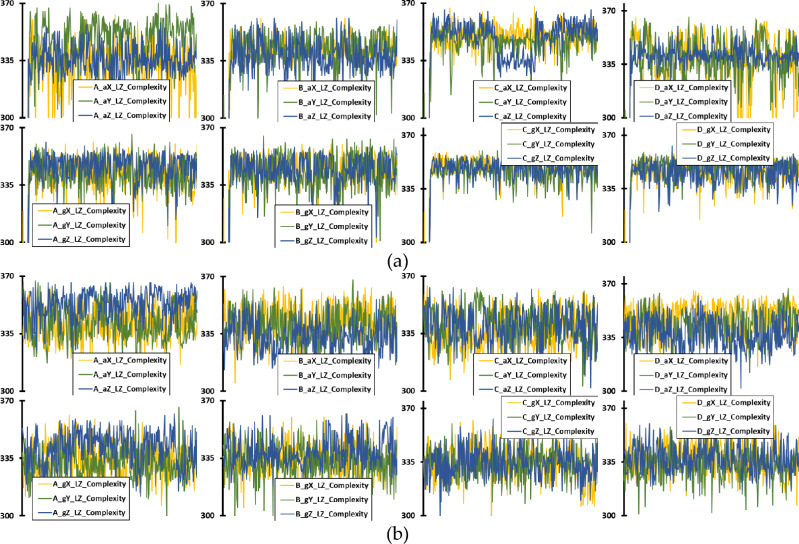
Time-domain LZC feature of accelerometer and gyroscope data for healthy UAV operation, with 345 instances in the x axis corresponding for an overall operation of 1 s, for: (**a**) Parrot Bebop 2; (**b**) 3DR Solo.

**Figure 10:**
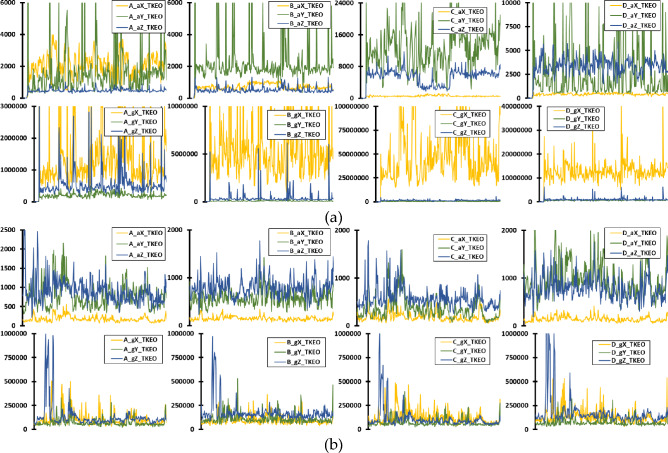
Time-domain TKEO feature of accelerometer and gyroscope data for healthy UAV operation, with 345 instances in the x axis corresponding for an overall operation of 1 s, for: (**a**) Parrot Bebop 2; (**b**) 3DR Solo.

Building upon the initial visualization of raw sensor data, Figures 8, 9, and 10 further explore the extracted features that encapsulate the underlying dynamics of UAV operation. Figure 8 illustrates the PE of accelerometer and gyroscope data across 345 instances, representing the nuanced, temporal complexity of UAV operations over one second for both UAV models. Similarly, Figure 9 presents the LZC feature, capturing the structural richness and unpredictability in the data patterns for the same duration and UAVs. Lastly, Figure 10 depicts the TKEO measurements, highlighting the instantaneous energy fluctuations within the UAV’s sensor data, again over a one-second period. Each figure—(a) for Parrot Bebop 2 and (b) for 3DR Solo—demonstrates the effectiveness of these advanced, non-traditional feature extraction techniques in characterizing the subtle yet critical variations in UAV operations, even when the UAVs are functioning normally. These visualizations underscore the potential of sophisticated analytical methods to detect minute anomalies that could precede actual operational failures, offering valuable insights into the robustness and sensitivity of the diagnostic process employed.

### Feature selection-testing and learning results

#### Important feature selection

**Figure 11:**
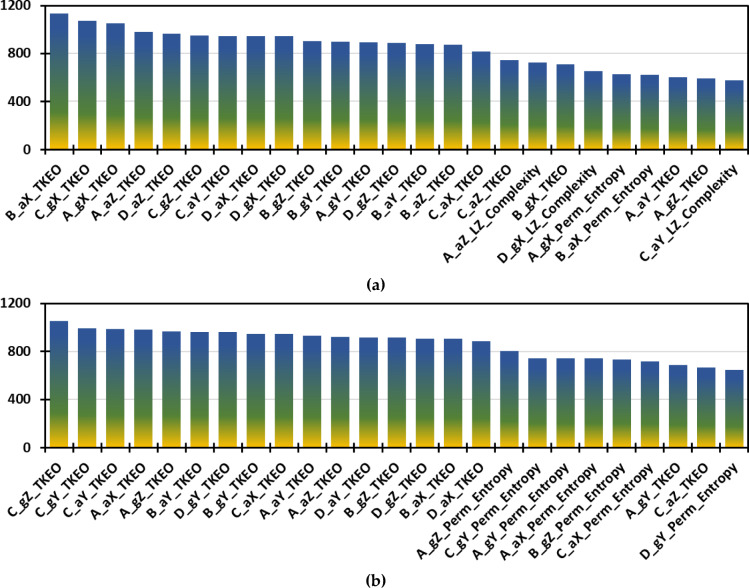
The χ^2^ important feature selection for the 72 features, showing the top 25 features: (**a**) Parrot Bebop 2; (**b**) 3DR Solo.

Figure 11 provides a compelling visualization of the χ^2^ feature selection process as it reveals the top 25 most influential features for UAV fault detection in both Parrot Bebop 2 (Figure 11a) and 3DR Solo (Figure 11b). A critical observation is the dominance of the TKEO across both UAV types, which is particularly prominent in high-scoring features such as the TKEO x-axis acceleration of the second propeller ‘B_aX_TKEO’ and the TKEO z-axis gyroscopic recordings of the third propeller ‘C_gZ_TKEO’ with scores of 1134.895 and 1052.151 respectively. This indicates that TKEO features generally carry more discriminative power in detecting anomalies compared to the other methodologies. Notably, LZC and PE are also present but to a lesser extent which suggests that while useful, they might not be as critical as TKEO for this specific application. For instance, ‘A_aZ_LZ_Complexity’ and ‘A_gX_Perm_Entropy’ appear further down the list with scores of 723.318 and 625.686. These results are demonstrating their lesser but still significant contributions to the feature set. This disparity highlights TKEO’s effectiveness in capturing dynamic changes in UAV propeller systems, potentially due to its sensitivity to energy fluctuations, which are crucial in fault detection scenarios.

Table 6 illustrates the results of the Taguchi method-tested features for UAV propeller fault diagnosis on the Parrot Bebop 2 drone to provide insight into how different combinations of PE, LZC, and TKEO influence diagnostic accuracy. Notably, the trial with all three features active, namely Trial 8, achieved the highest accuracy of 98.7% which indicates a synergistic effect when integrating these features. This is underscored by the S/N ratio, which assumably reflects a high robustness in signal quality due to the comprehensive feature integration. In contrast, the trials with fewer features enabled generally exhibit lower performance that suggests each feature contributes uniquely to fault detection. Specifically, trials with only a single feature enabled (Trials 1, 6, and 7) likely resulted in significantly lower accuracy and S/N ratios, underscoring the inadequacy of relying on a singular dimension of data for complex fault diagnosis scenarios. This pattern affirms the necessity of a multi-faceted approach in feature extraction for enhancing diagnostic precision in UAV systems.

**Table 6:** Results of the Taguchi-tested features of the Parrot Bebop 2 drone.

Trial	PE	LZC	TKEO	Accuracy (%)	S/N ratio (dB)
1	0	0	0	86.3	20.5
2	0	1	1	92.5	24.7
3	1	0	1	93.1	25.2
4	1	1	0	94.7	26.8
5	1	0	0	90.0	22.5
6	0	1	0	89.2	21.8
7	0	0	1	91.6	23.9
8	1	1	1	98.7	30.0

#### UAV fault type diagnostical modeling

The evaluation of DNN performance before and after the implementation of χ^2^ feature selection underscores significant improvements across various metrics, as demonstrated in Tables 7 and 8. Before χ^2^ feature selection, the DNN achieved accuracies of 97.8% and 99.1% for the Parrot Bebop 2 and 3DR Solo UAV types, respectively. However, with the χ^2^-selected features, these accuracies improved to 98.7% and 99.6%. This enhancement is particularly noteworthy for the Parrot Bebop 2, which saw a 0.9% increase in accuracy. Similarly, other performance metrics such as F1-Score, Precision, and Recall for the Parrot Bebop 2 also rose by 0.9%, highlighting the effectiveness of χ^2^ feature selection in isolating the most impactful features for fault detection in UAV propellers.

**Table 7:** Results of the classification DNN using all features.

UAV type processed dataset	Instances per dataset	Sampling	Repeat train/test	Test/train percentages	Accuracy	F1-score	Precision	Recall
Parrot Bebop 2	345	Random	5	25%, 75%	97.8%	97.8%	97.8%	97.8%
3DR Solo	345	Random	5	25%, 75%	99.1%	99.1%	99.1%	99.1%

**Table 8:** Results of the classification DNN using χ^2^-selected features.

UAV type processed dataset	Instances per dataset	Sampling	Repeat train/test	Test/train percentages	Accuracy	F1-score	Precision	Recall
Parrot Bebop 2	345	Random	5	25%, 75%	98.7%	98.7%	98.8%	98.7%
3DR Solo	345	Random	5	25%, 75%	99.6%	99.5%	99.5%	99.5%

**Figure 12:**
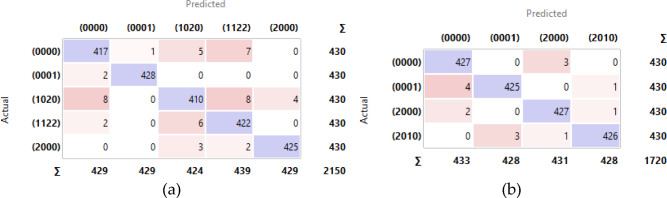
Confusion matrix for the utilized DNN without χ^2^ employment: (**a**) Parrot Bebop 2; (**b**) 3DR Solo.

**Figure 13:**
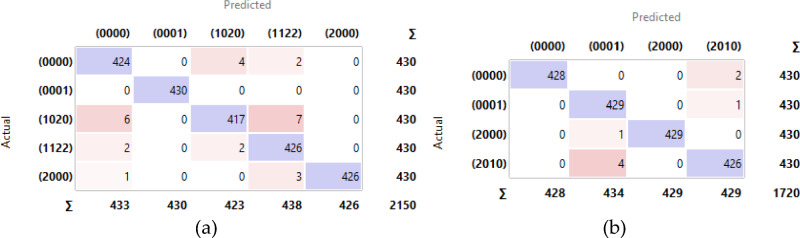
Confusion matrix for the utilized DNN with χ^2^ employment: (**a**) Parrot Bebop 2; (**b**) 3DR Solo.

Further analysis using the confusion matrices depicted in Figures 12 and 13 reveals more detailed insights. The confusion matrix for the Parrot Bebop 2 without χ^2^ feature selection (Figure 12a) shows some misclassifications, which were notably reduced in the scenario with χ^2^ feature selection (Figure 13a). This reduction in error is mirrored in the results for the 3DR Solo UAV, where the application of χ^2^-selected features led to almost perfect classification metrics (Figure 13b). These results validate the hypothesis that employing advanced feature selection techniques can significantly enhance the predictive performance of fault detection systems in UAVs. The increased precision and recall, alongside improved overall accuracy, confirm the robustness of the proposed method in effectively diagnosing UAV propeller faults, setting a promising benchmark for future research in UAV maintenance protocols.

### Comparative analysis

Table 9 provides a comparative analysis with recently and previously published studies on UAV fault diagnosis, with a highlight on the advancements made in this research. The current study, utilizing the PADRE repository with innovative features of PE, LZC, and TKEO, achieves a remarkable diagnostic accuracy of over 97.8%. This is notably higher compared to other studies listed, which predominantly utilized standard statistical features and achieved accuracies ranging from 91.0% to 96.0%. The use of advanced, non-traditional features in conjunction with artificial intelligence in this study not only underscores the efficacy of integrating sophisticated analytical techniques but also sets a new benchmark in the precision of UAV propeller fault diagnosis.

**Table 9:** Comparative analysis with previously published studies.

References/study	Utilized dataset	Utilized features	Use of AI	Resulted accuracy
^41^	DJI Mini Combo 2 Vibrational Dataset	Standard statistics	Yes	Over 91.0%
^42^	The Falcon V5 UAV Acoustic Dataset	Standard statistics and spectrum	Yes	Over 91.8%
^43^	DJI Mini Combo 2 Vibrational Dataset	Standard statistics, spectrum, and multiresolution	No	N/A
^44^	DJI Mini Combo 2 Vibrational Dataset	Standard statistics	Yes	Over 96.0%
^45^	DJI Mini Combo 2 Vibrational Dataset	Standard statistics	Yes	Over 92.52%
^46^	DJI Mini Combo 2 Vibrational Dataset	Standard statistics, filtering, and multiresolution	No	N/A
^21^	PADRE repository	Standard statistics	Yes	Over 95.1%
^47^	DJI Mini Combo 2 Vibrational Dataset	Standard statistics	Yes	Over 94.0%
This study	PADRE repository	PE, LZC, and TKEO	Yes	Over 97.8%

## Conclusions and future directions

This study successfully demonstrated the efficacy of non-traditional feature extraction methodologies in enhancing the fault detection capabilities for UAV propellers. By employing PE, LZC, and particularly TKEO, the research established a robust framework for accurate fault diagnostics. The extensive analysis concluded that TKEO features were the most prominent in feature selection, frequently appearing in the top-ranked features, thereby their numbers underscore their critical role in the model’s predictive performance. Interestingly, the use of Taguchi method proved the effectiveness of all three features. Notably, the implementation of χ^2^ feature selection significantly boosted the classification accuracy, where the most substantial improvement observed was a 0.9% increase for the Parrot Bebop 2 model as it reached an accuracy of 98.7%. This enhancement in performance metrics firmly supports the integration of advanced feature extraction techniques in the predictive maintenance of UAV systems. A comparative analysis was also conducted at the end of the results to demonstrate the potential of untraditional vibration analysis features over traditional ones.

The promising results from this research pave the way for several future studies. First, the exploration of additional advanced feature selection methods could potentially uncover even more effective feature sets, optimizing fault detection systems further. Furthermore, integrating a larger and more varied dataset could validate the generalizability and robustness of the current findings across different UAV models and environments. Another exciting avenue would be the application of real-time data processing techniques to enable on-the-fly fault detection, which is crucial for operational UAVs. Lastly, a deeper investigation into the interactions between different feature extraction methods could provide insights into creating a more synergistic approach. This would potentially lead to even higher performance metrics in UAV fault diagnostics.

## Data Availability

Data is provided within the manuscript or supplementary information files.
